# Role of 5-HT_3_ Receptors in the Antidepressant Response

**DOI:** 10.3390/ph4040603

**Published:** 2011-04-07

**Authors:** Cécile Bétry, Adeline Etiévant, Chris Oosterhof, Bjarke Ebert, Connie Sanchez, Nasser Haddjeri

**Affiliations:** 1 University of Lyon, F-69008, Lyon, France; 2 Laboratory of Neuropharmacology, Faculty of pharmacy, University of Lyon 1, F-69008, Lyon, France; 3 H. Lundbeck A/S, Ottiliavej 9, DK-2500 Valby, Denmark; 4 Lundbeck Research USA, Inc., Paramus, NJ 07652, USA

**Keywords:** 5-HT3 receptors, depression, new therapeutics

## Abstract

Serotonin (5-HT)_3_ receptors are the only ligand-gated ion channel of the 5-HT receptors family. They are present both in the peripheral and central nervous system and are localized in several areas involved in mood regulation (e.g., hippocampus or prefrontal cortex). Moreover, they are involved in regulation of neurotransmitter systems implicated in the pathophysiology of major depression (e.g., dopamine or GABA). Clinical and preclinical studies have suggested that 5-HT_3_ receptors may be a relevant target in the treatment of affective disorders. 5-HT_3_ receptor agonists seem to counteract the effects of antidepressants in non-clinical models, whereas 5-HT_3_ receptor antagonists, such as ondansetron, present antidepressant-like activities. In addition, several antidepressants, such as mirtazapine, also target 5-HT_3_ receptors. In this review, we will report major advances in the research of 5-HT_3_ receptor's roles in neuropsychiatric disorders, with special emphasis on mood and anxiety disorders.

## 1. Introduction

Major depression is one of the most frequent psychiatric disorders. In the United States, the lifetime prevalence of this disease is about 16% [1]. It is an important public health problem since major depression induces disability, poor quality of life, economic burden or suicide. According to the monoaminergic theory of depression, deficiencies or imbalances in monoamine neurotransmitters, *i.e.*, serotonin (5-HT), noradrenaline (NA) and dopamine (DA), are involved in the pathophysiology of this disease. Development of antidepressants in the last five decades has been mainly based on this hypothesis. In addition, it is now well established that both pathophysiology of depression and effect of antidepressant treatments involve neuroplasticity (e.g., hippocampal neurogenesis, expression of the brain-derived neurotrophic factors (BDNF)) and hypothalamic pituitary-adrenal (HPA) axis modulation [2,3]. Nevertheless, despite their large range, pharmacotherapy treatments of depression remain unsatisfactory [4]. Firstly, patients often receive several antidepressant agents before responding to a treatment and only about 65% experience some degree of therapeutic response [5]. Secondly, treatment for weeks or months is necessary before a therapeutic response is achieved; this therapeutic delay is critical since it can be associated with increased risk of suicide [6].

New pharmacological strategies have emerged to improve efficacy and reduce the time for antidepressants to act. Preclinical studies have suggested that targeting specific 5-HT receptors with selective agonist or antagonist drugs may enhance the antidepressant response and reduced its delay compared to currently used antidepressants. Both the 5-HT_1A_ receptor antagonist pindolol and 5-HT_1A_ receptor agonists, e.g., buspirone, have been largely investigated both in clinical and in preclinical studies in combination with antidepressants with some significant effects [7,8]. Moreover, the beneficial effect of atypical antipsychotics, such as quietapine, in combination with antidepressants in depression may be partly due to their 5-HT_2A_ receptor targeting [9,10]. More recently, preclinical studies suggested that 5-HT_4_ receptor agonists are putative antidepressants with a fast onset of action [11,12]. Similarly, 5-HT_7_ receptor antagonists may improve efficacy and delay of action of classical antidepressants [13]. In this review, we will report major advances in the discovery of 5-HT_3_ receptor roles, with special emphasis on the potential role of 5-HT_3_ receptor antagonism in mood and anxiety disorders. Although their clinical use mainly concerns chemotherapy-induced emesis, several preclinical and clinical studies suggest their relevance in treatment of psychiatric disorders. After a brief description of the structure and the physiological function of 5-HT_3_ receptors, we will describe their brain distribution. Then, we will review the effects of 5-HT_3_ receptor agonists and antagonists in brain areas involved in the pathophysiology of depression. Finally we will summarize several behavioral non-clinical studies and clinical studies revealing its role in the antidepressant response.

## 2. Structure and Function of 5-HT_3_ Receptors in the Central Nervous System

### 2.1. Structure of 5-HT_3_ Receptors

The 5-HT_3_ receptors are the only ionotropic or ligand gated ion channel of the 5-HT receptor family [14]. They are members of the Cys-loop superfamily of ligand-gated ion channels [15] as are nicotinic acetylcholine (nAch) receptors and gamma-aminobutyric acid-type-A (GABA)_A_ receptors. Thus, they are composed of five subunits forming a cylinder that can be crossed by cations [16]. In rats and mice, two subunits have been cloned: 5-HT_3A_ [15] and 5-HT_3B_ [17] receptor subunits which can be arranged in homomeric 5-HT_3A_ receptors and heteromeric 5-HT_3A_/5-HT_3B_ receptors [17]. Three others subunits have been also described in multiple mammalian species but not in rodents: 5-HT_3C_, 5-HT_3D_ and 5-HT_3E_ [18-20] which probably form only heteromeric receptors with 5-HT_3A_ receptor subunits.

### 2.2. Location of the 5-HT_3_ Receptor in the CNS

Using different methods including autoradiography, immunohistochemistry and *in situ* hybridization, the 5-HT_3_ receptor distribution has been largely described with some differences between species. 5-HT_3_ receptors are expressed both in peripheral and central nervous system. In the periphery, 5-HT_3_ receptors are located on pre- and postganglionic neurons from autonomic nervous system and on neurons of the sensory and enteric nervous system [21]. In the central nervous system, 5-HT_3_ receptor density appeared low compared to others 5-HT receptors [14,22]. In all species, including humans, the most important densities of 5-HT_3_ receptors seems to be found in the hindbrain in particular in the dorsal motor nucleus of the vagus nerve, in the nucleus of the tractus solitaries and in the area postrema [22-32]. These brain areas are involved in the vomiting reflex explaining the relevance of 5-HT_3_ receptor antagonist in chemotherapy-induced emesis [14]. Significant densities of 5-HT_3_ receptors have also been described in the spinal cord [33,34].

Compared to the hindbrain, the density of 5-HT_3_ receptors in the forebrain is lower. Nevertheless, significant levels of these receptors have been found in brain areas involved in the pathophysiology of depression with densities varying across species.

In non primate mammals, 5-HT_3_ receptors are found in the limbic areas including the amygdala, hippocampus, nucleus accumbens and in the superficial layers of the cerebral cortex, *i.e*., the frontal parts (in particular cingulate, prelimbic and infralimbic areas and primary and secondary motor areas), entorhinal and temporal cortex [25,27-29,31,33,35-38]. In addition, some studies have demonstrated that, they are also present at low densities in the dorsal raphe nucleus, striatum, substantia nigria and nucleus accumbens [25,28-30,38].

In the marmoset forebrain, 5-HT_3_ receptors are found in medial habenula nucleus and the hippocampus [32]. In the human forebrain, 5-HT_3_ receptors are found essentially in limbic structures such as amygdala, hippocampus, nucleus accumbens and striatum whereas only low levels of 5-HT_3_ receptors have been found in cortex [23,28,39-42].

A significant amount of 5-HT_3_ receptors are localized on presynaptic nerve fibers and terminals [30,43-46]. Indeed, in cortical areas, amygdala [47] and in striatum [48,49], 5-HT_3_ receptors are essentially located presynaptically whereas in hippocampus, the postsynaptic receptors are predominant [47].

### 2.3. Pharmacology and Physiology of 5-HT_3_ Receptors

5-HT_3_ receptors are permeable to Na^+^, K^+^ and Ca^2+^ [50,51]. Stimulation leads to opening of the ion channel, inducing a rapid membrane depolarization mediated by cation flow [52,53]. The function of 5-HT_3_ receptors depend on their localization: nerve-terminal 5-HT_3_ receptors activation leads to release of various neurotransmitters such as 5-HT, DA or GABA [54], whereas the activation of postsynaptic 5-HT_3_ receptors is involved in fast synaptic transmission [55,56]. Pre- and postsynaptic receptors are associated with specific characteristics including a different hill coefficient, different single channel conductances, different kinetics, a different re-sensitization time-course [17,57,58]. In particular, presynaptic 5-HT_3_ receptor displays a high permeability to Ca^2+^ [45,46,48,59], whereas postsynaptic receptors display a lower permeability to Ca^2+^ compared to Na^+^ and K^+^ [50,60]. Similarly, homomeric 5-HT_3A_ receptors are equally permeable to monovalent and divalent cations whereas heteromeric 5-HT_3_ receptors have a lower permeability to Ca^2+^ [17,61,62]. Moreover, heteromeric receptors display faster activation and deactivation kinetics than homomeric receptors [62]. *In vitro* studies are generally performed in cultured cells expressing only homomeric 5-HT_3_ receptors [61] which can explain several differences obtained by *in vitro* and *in vivo* studies.

5-HT_3_ receptor agonists and antagonists present different affinity and efficacy depending on the structure of 5-HT_3_ receptors, *i.e.*, heteromeric or homomeric [17,58,61] (Table 1). 5-HT_3_ receptor agonists seem not have clinical interests. Frequently used preclinical tool agonists are 1-(*m*-chlorophenyl)-biguanide (mCPBG) and 2-methyl-5-HT (2-Me-5-HT) which do not cross the blood brain barrier. These compounds are not selective for 5-HT_3_ receptors since mCPBG has notable affinity for the DA transporter [63] while 2-Me-5-HT has notable affinity for other 5-HT receptor subtypes [64,65]. SR57227A is the mostly used 5-HT_3_ receptor agonist that crosses the blood brain barrier [66].

In comparison to 5-HT3 receptor agonists, many 5-HT_3_ receptor antagonists have been developed and they are widely used in the clinic. The main therapeutic use of 5-HT_3_ receptor antagonist is for chemotherapy-induced emesis [67]. However, other therapeutic uses of 5-HT_3_ receptor antagonist have been suggested e.g. pain, addiction and psychiatric disorders [68]. In regard with psychiatric disorders, 5-HT_3_ receptor antagonists present anxiolytic and antidepressants effect (see below) but they may also have antipsychotic effect even if data are yet controversial [21,69]. The 5-HT_3_ antagonists may be identified by the suffix setron. Different drugs belonging to the “setron class” are used in the clinic: MDL 73,147EF (dolasetron), GR38032F (ondansetron), BRL 43694 (granisetron), ICS 205-939 (tropisetron), palanosetron listed according to ascending receptor binding affinity (7.73 to 10.45 nM) [67]. Other “setrons” have been used in preclinical studies including DAU 6215 (itasetron), BRL-46470A (ricasetron), MDL 72222 and LY277359 (zatosetron).

Curiously, 5-HT_3_ receptor agonist or antagonist responses are frequently associated with a bell-shaped dose-response curve and this is the case for both clinical and preclinical studies. Generally, the maximum effect is typically observed at very low dose, in the microgram range, while higher doses are ineffective [21]. For example, such responses were observed in the rat learned helplessness [70], the forced swim test and the tail suspension test [71] as well as in the induction of theta rhythms [72]. The inverse dose-response relation of 5-HT_3_ receptor agonists may be explained by receptor desensitization. Receptor desensitization is involved in mediation of short-term plasticity of synapses. Such mechanisms seem to be involved in regulation of 5-HT_3_ receptors activity in cultured cells [52]. This desensitization can be explained by receptor internalization. In fact, when cells expressing 5-HT_3_ receptors in the plasma membrane were activated by the 5-HT_3_ receptor agonist, mCPBG, a decrease of 5-HT_3_ receptor density was observed after a few minutes [73]. Internalization can be prevented by the 5-HT_3_ receptor antagonist ondansetron [74]. Interestingly, it has been recently demonstrated that the 5-HT_3_ receptor antagonist palanosetron can also induce 5-HT_3_ receptor internalization and cause prolonged inhibition of receptor function whereas ondansetron and granisetron do not [75]. Moreover, MDL7222 fails to induce internalization of 5-HT_3_ receptors since a chronic treatment with this compound does not alter sensitivity to a 5-HT_3_ receptor agonist [76]. Thus, internalization of 5-HT_3_ receptors is not sufficient to explain the bell-shaped dose-response curve observed under various conditions [21] brought forward hypothesizes of steric hindrance at higher concentrations, different effect on hetero/homoreceptors inducing an effect on some receptors at a low concentration and another effect at higher concentration explaining a heterogeneous response depending on concentration.

## 3. Effect of 5-HT_3_ Receptor Ligands on Neuronal Firing and Neurotransmitter Release

### 3.1. Interaction with 5-HT Systems: Effect in Dorsal Raphe Nucleus

Dorsal raphe nucleus is the brain structure with the highest density of 5-HT cell bodies. Electrophysiological studies demonstrated that the 5-HT_3_ receptor agonist, phenylbiguanide, has no effect on the dorsal raphe nucleus 5-HT cell firing whereas the other 5-HT_3_ receptor agonist 2-Me-5-HT has an inhibitory action [65,77]. However, the interpretation of these findings are difficult as there are reports showing that a 5-HT_1A_ receptor antagonist can prevent the suppressant effect of a 5-HT_3_ agonist [77], and others reporting a lack of effect of a 5-HT_3_ receptor antagonist and 5-HT_1A_ antagonist to reverse the effect of the agonist [65]. Furthermore, both *in vivo* and *in vitro*, studies of various 5-HT_3_ receptor antagonists including ondansetron and zacopride report no significant effect on the dorsal raphe nucleus 5-HT cell firing [65,77]. Interestingly, it has been shown *in vitro* that 2-Me-5-HT induces a release of 5-HT in raphe nuclei slices both under basal conditions and also after stimulations [78]. This release of 5-HT explains the decrease of firing observed with 5-HT_3_ receptor agonists since dorsal raphe nucleus neurons mediate inhibitory effects at 5-HT_1A_ autoreceptors. The 5-HT release induced by 5-HT_3_ receptor agonists is also not specific in basal conditions: it can be antagonized by fluoxetine and not by 5-HT_3_ receptor antagonists whereas it is reversed in stimulated conditions by ondansetron [78]. Thus, it seems that in dorsal raphe nucleus, there is no detectable 5-HT tone at 5-HT_3_ receptors and that contradictory results obtained in basal conditions can be explained by the poor selectivity of 5-HT_3_ receptors agonists.

### 3.2. Interaction with GABAergic Interneurons

The role of GABAergic neurotransmission in depression is a relative new area of research [79]. Changes in GABAergic function has been observed in animal model of depression [80] and GABA receptor agonists seems to present antidepressant-like properties [81]. Moreover, serotonergic neurons connect mainly GABAergic interneurons suggesting a strong interaction between the two systems [82,83]. GABAergic interneurons expressing 5-HT_3_ receptors have been essentially detected in the hippocampus and prefrontal cortex [84].

#### 3.2.1. Hippocampus

Since antidepressants modulate serotoninergic and noradrenergic systems in the hippocampus, it has been suggested that the hippocampus is involved in the pathophysiology of depression [85]. The activity of hippocampus is strongly regulated by GABAergic interneurons. These inhibitory neurons are notably controlled by 5-HT inputs [86]. Hippocampus is one of the forebrain areas in which 5-HT_3_ receptors are localized both post- and pre-synaptically. In the hippocampus, 5-HT_3_ messenger ribonucleic acids (mRNAs) and proteins are essentially localized on interneurons [87]. Interestingly, more than 50% of the hippocampal GABAergic interneurons express 5-HT_3_ receptors [88]. 5-HT_3A_ and 5-HT_3B_ mRNA and protein subunits are present in the human hippocampus [17,89].

*In vivo*, infusion of the 5-HT_3_ receptor agonist 2-Me-5-HT induces 5-HT release in the hippocampus [78,90]. Interestingly, after an 10 µM infusion of 2-Me-5-HT the 5-HT level rapidly goes back to baseline while with after 1µM, the effect is maintained for 45 minutes [90]. Similarly, *in vitro* electrically-evoked 5-HT release was enhanced by a 5-HT_3_ receptor agonist [65]. These effects are prevented by 5-HT_3_ receptor antagonists while the antagonist has no effect on basal 5-HT release [65,90]. Thus, hippocampal 5-HT_3_ receptors seem not to be tonically activated. Nevertheless, even if 5-HT_3_ receptor antagonists have no basal effect on the hippocampal 5-HT release, they can increase 5-HT levels induced by the selective serotonin reuptake inhibitor (SSRI) paroxetine [91].

*In vivo*, 2-Me-5-HT reduces the hippocampal transmission by reducing the amplitude of evoked field potentials and this effect is blocked by the 5-HT_3_ receptor antagonist ricasetron [92] whereas *in vitro* 2-Me-5-HT has no significant electrophysiological effect on CA1 pyramidal cells at basal state [93,94]. Nevertheless, it reduces clearly hippocampal plasticity by decreasing long term potentiation (LTP) and long term depression (LTD), evoked by stimulation of Schaffer collaterals [93,94]. Conversely, 5-HT_3_ receptor antagonists induce an increase in LTP induction associated with behavioral increase of memory [72,94,95]. Granisetron and a GABA receptor antagonist prevent this effect whereas 5-HT_1_ or 5-HT_2_ receptor antagonists have no effect [93, 94]. As mentioned above, 5-HT_3_ receptor stimulation in hippocampus induces a release of 5-HT and it is well-known that 5-HT has an inhibitory effect on hippocampal LTP [96] which may explain the suppressant effect of a 5-HT_3_ receptor agonist on LTP. Interestingly, a 5-HT_3_ receptor antagonist prevented this 5-HT-mediated blockage of LTP [93,96,97]. Firing rate of *in vivo* CA1 pyramidal cells that are iontophoretically activated by glutamate is suppressed by SR57227A and 2-me-5-HT, whereas the 5HT3 receptor antagonist ricasetron prevents this suppressant effect [92].

In hippocampus slices, 5-HT increases the frequency and amplitude of spontaneous GABAergic inhibitory postsynaptic potentials (IPSPs), an effect that is inhibited by 5-HT_3_ receptor antagonists [43,59,98-100]. A 5-HT_3_ receptor agonist acts similar to the endogenous ligand on IPSPSs [59]. Thus, 5-HT_3_ receptor agonists depolarize hippocampal interneurons [101,102] and induce GABA release by opening of voltage-gated Ca^2+^ channels [59,100]. This effect has a rapid onset and desensitization [59]. These inhibitory GABAergic interneurons regulate function of both hippocampal CA1 pyramidal cells and dentate gyrus cells [101].

5-HT_3_ receptor agonists reduce both hippocampal transmission and plasticity and 5-HT_3_ receptor antagonists inhibit this effect. Stress reduces LTP and it has been suggested that alterations of hippocampal LTP may have a role in the etiology of depression. Yet, current antidepressants inhibit LTP [3]. Hence, addition of a 5-HT_3_ receptor antagonist may prevent the antidepressant-induced LTP decrease and thereby improve efficacy of current antidepressants and including memory deficits.

#### 3.2.2. Prefrontal Cortex

Prefrontal cortex is frequently metabolically overactive in treatment-resistant depression and clinical improvement after pharmacotherapy, psychotherapy or limbic leucotomy is correlated with decreases in its metabolic activity [103]. The majority of 5-HT_3_ receptors in the prefrontal cortex cells are co-expressed with glutamic acid decarboxylase (GAD), a marker of GABAergic neurons [35]. In the neocortex, more than 90% of cells expressing 5-HT_3_ receptors are GABAergic neurons [84,88]. In prefrontal cortex, only 5-HT_3A_ receptor subunit seems to be expressed [104]. GABAergic interneurons expressing 5-HT_3_ receptors are also co-localized with cholecystokinin (CCK) and vasoactive intestinal peptides but not somatostatin, and may express the Ca^2+^ binding proteins calbindin and calretinin but not parvalbumin [88,104]. Similarly in rhesus monkeys, the majorities of 5-HT_3_ receptor positive cortical cells are GABAergic neurons and generally co-expresses substance P receptors. These neurons can also express calbindin or calretinin [105].

*In vitro* 2-Me-5-HT enhances the 5-HT release in rat and guinea pig frontal cortex, an effect inhibited by 5-HT_3_ receptor antagonists [65,106]. Applied by microiontophoresis, they induce an inhibitory effect on the firing activity of medial prefrontal cortex cells [65,107-110]. Some investigators found that the effect is specific, *i.e.*, it is blocked by selective 5-HT_3_ receptor antagonists and not by 5-HT_1_ and 5-HT_2_ receptor antagonist and GABA-A receptor antagonists [107-110], whereas for others report that the effect is not blocked by a 5-HT_3_ receptor antagonist but by a 5-HT_1A_ receptor antagonist [65]. Similarly, 5-HT_3_ receptor agonists depressed firing of glutamate-activated quiescent medial prefrontal cortex cells [109]. Moreover, SR57227A depressed *N*-Methyl-d-aspartate (NMDA)-evoked membrane depolarization, action potentials, and inward current in the rat medial prefrontal pyramidal cells, an effect that was reversed by a 5-HT_3_ receptor antagonist [111]. Interestingly, acute administration of an SSRI, like citalopram, increases 5-HT levels in prefrontal cortex and ondansetron can enhance this increase [91].

**Figure 1:**
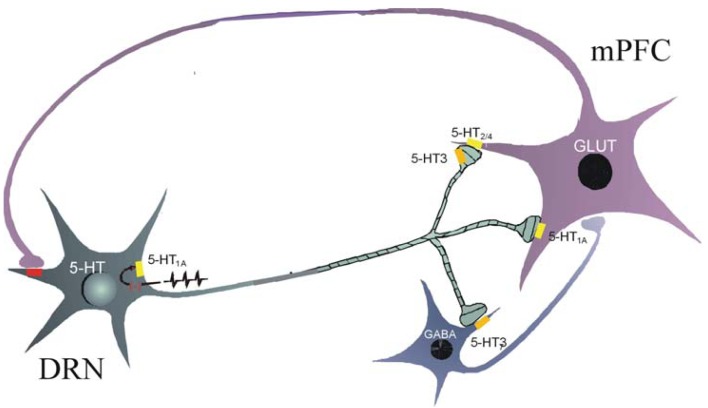
Hypothetic model of the mechanism of action of 5-HT_3_ receptor agonists on the dorsal raphe nucleus-prefrontal cortex loop. Cortical 5-HT_3_ receptors seem to be localized both presynaptically on 5-HT neurons and postsynaptically on GABAergic interneurons. Stimulation of dorsal raphe nucleus induces a cortical release of 5-HT. Thus, released 5-HT may target postsynaptic 5-HT_3_ receptors inducing an activation of GABAergic interneurons. These interneurons inhibit pyramidal cells that reduce 5-HT dorsal raphe neuronal firing rate. 5-HT receptors are represented in yellow except 5-HT_3_ receptors in orange and ionotropic glutamatergic receptors are represented in red. DRN, dorsal raphe nucleus, mPFC, medial prefrontal cortex, GABA, gamma aminobutyric acid, GLUT, glutamate.

The suppressant effect on the firing activity of medial prefrontal cortex cells induced by a 5-HT_3_ receptor agonist is mediated by an indirect mechanism. A 5-HT_3_ receptor agonist induces a direct inward current with a rapid desensitization of interneurons but has no effect in cultured pyramidal cells [112]. Thus, it appears that 5-HT_3_ receptor agonists increase inhibitory postsynaptic currents (IPSCs) in cortical pyramidal neurons via stimulation of 5-HT_3_ receptors located on inhibitory GABAergic interneurons [104,112]. In contrast to 5-HT_2A_ receptors which are localized both in inhibitory and excitatory neurons, 5-HT_3_ receptors have only an inhibitory effect in cortex [105,112]. As a consequence, a pulse of 5-HT is inhibitory while prolonged presence of 5-HT can induce enhancement of transmission because of 5-HT_2A_ receptor activation and 5-HT_3_ receptor desensitization [112]. There is a regulatory loop between raphe dorsalis and prefrontal cortex since stimulation of dorsal raphe induces an excitation of slow-spiking GABAergic neurons in prefrontal cortex inducing a suppression of cortical cells, an effect blocked by a 5-HT_3_ receptor antagonist [35,108] (Figure 1).

### 3.3. Interaction with the Dopamine System

The dopamine system can be divided into three pathways. The nigrostriatal dopaminergic pathway consists of the substantia nigra pars compacta (SNc, A9) and their associated efferent targets in the dorsal striatum. The mesolimbic dopaminergic pathway contains A10 dopaminergic neurons located within the ventral tegmental area (VTA) and their associated efferent targets in the ventral striatum including nucleus accumbens and limbic structures (e.g., amygdala and hippocampus). The mesocortical dopaminergic pathway consists of the A10 dopaminergic neurons and their associated efferent targets in the prefrontal cortex. It has been suggested that abnormalities of dopaminergic neurotransmission have been implicated in the pathogenesis of affective disorders [113,114]. In particular, several studies have suggested that the mesolimbic pathway can be altered in mood disorders. For example, stress, in animal models of depression, activates VTA DA neurons and their limbic efferent targets [115]. Moreover, the DA system is now one of the targets of depression treatment, for example drugs initially used in schizophrenia like aripiprazole or quetiapine are now used in depressed patients [8].

#### 3.3.1. Substantia nigra compacta (SNc)

Acute administration of ondansetron, zatosetron or itasetron fails to modify the number of DA neurons that are spontaneously active in SNc (Table 2). Similarly, 21-day-chronic treatment with 5-HT_3_ receptor antagonists has no effect, except for zatosetron at 0.1 mg/kg/day [116] and dolasetron at 5 mg/kg/day [117] which both decreased number of DA cells that are spontaneously active(Table 2). Moreover, 5-HT_3_ receptor antagonists fail to affect sensibility to the D2-like receptor agonist apomorphine in SNc [118].

#### 3.3.2. Striatum

Infusion of striatal slices with 5-HT_3_ receptor agonists, including 5-HT, produce an increase in DA release, an effect that is blocked by a 5-HT_3_ receptor antagonist [119-122]. Similarly, *in vivo* studies in rats demonstrated that endogenous 5-HT stimulates release of 5-HT in striatum and that this effect is partially blocked by a 5-HT_3_ receptor antagonist [123]. Notably, these data are controversial and in some studies the release induced by 5-HT_3_ receptor agonists is not found to be 5-HT_3_ receptor specific [124,125] wheras in other studies 5-HT_3_ receptor agonists fail to modify basal striatal DA release [126]. 5-HT_3_ receptor antagonists have failed to induce DA release in the striatum both *in vitro* and *in vivo* [127-132]. Dorsal raphe nucleus stimulation produces a DA decrease in the striatum which is not modified by 5-HT_3_ receptor antagonists [133]. In fact, 5-HT_3_ receptor-induced DA release may be effective only when both DA and 5-HT tone is increased [134].

#### 3.3.3. Ventral tegmental area (VTA)

Local infusion of mCPBG induces an increase of extracellular levels of DA in VTA slices, an effect that is inhibited by a 5-HT_3_ receptor antagonist and depending on Ca^2+^ [76,135,136]. On the other hand, acute administration of 5-HT_3_ receptor antagonists ricasetron and itasetron has no effect on the firing of VTA DA neurons [118,137,138] and do not affect sensitivity to the D2-like receptor agonist apomorphine [118]. Nevertheless, clorgyline, a monoamine oxidase inhibitor (MAOI), inhibits VTA DA firing rate and ondansetron reverses this effect [138]. Effects of 5-HT_3_ receptor antagonists on the number of DA cells per track are rather variable. Both after acute and chronic treatment, 5-HT_3_ receptor antagonists have no effect or induced an increase or a decrease of the number of DA cells that are spontaneously active. These results depend on the 5-HT_3_ receptor antagonist used and the dose used (Table 2). Moreover, when a 5-HT_3_ receptor antagonist produces a decrease of the number of DA cells/track after a chronic treatment, apomorphine can or cannot reverse this effect [118].

#### 3.3.4. Nucleus accumbens

*In vivo* and *in vitro* local administrations of a 5-HT_3_ receptor agonist induced an increase of extracellular DA release in nucleus accumbens, an effect which is blocked by a 5-HT_3_ receptor antagonist [76,139-141], suggesting a modulation of DA function by presynaptic 5-HT_3_ receptors in this area [142]. Moreover, in 5-HT_3_ receptors over-expressed mice, the DA release induced by a 5-HT_3_ receptor agonist was increased compared to wild type [143]. Similarly, dorsal raphe nucleus stimulation increases DA release in nucleus accumbens which is attenuated by a 5-HT_3_ receptor antagonist [133]. Interestingly, in an animal model of depression, namely the Flinder sensitive Line rats, 5-HT_3_ receptor agonists do not increase DA levels in nucleus accumbens while a chronic antidepressant treatment restore the DA increase in response to agonist [113]. Acute administration of a 5-HT_3_ receptor antagonist does not modify the rate of DA release in nucleus accumbens [130,144] while chronic treatment with the 5-HT_3_ receptor antagonist MDL72222 decreases the extracellular concentration of DA in the nucleus accumbens [76,131]. This effect is not due to an alteration of sensitivity of 5-HT_3_ receptors since the DA release induced by a 5-HT_3_ receptor agonist is not altered after MDL72222 treatment [76]. Finally, local administration of a 5-HT_3_ receptor antagonist in nucleus accumbens has an anxiolytic-like effect in different behavioral tests such as open-field, Vogel conflict test and light-dark exploration [145,146].

### 3.4. Interaction with Other Neurotransmitters

There is less data concerning other neurotransmitters release and this relevance of this data for depression is less clear data. Nevertheless, there is some evidence that 5-HT_3_ receptors may also induce a release of glutamate from nerve terminals [148-150]. Also, there might be a role for acetylcholine (Ach), since 5-HT_3_ receptor activation inhibits cortical release of Ach. It is not clear, however, whether this is a direct effect. Moreover 5-HT_3_ receptor antagonist induced an increase of cortical Ach release, an effect that was potentiated by GABA receptor antagonists. This has led to the suggestion that the augmented ACh release by 5-HT_3_ antagonists causes a blockade of GABA-mediated inhibition on cholinergic neurons [14,151]. Finally, some studies evoked an increase of noradrenaline induced by activation of 5-HT_3_ stimulation but some others data are controversial and this effect seems not specific [85].

## 4. 5-HT_3_ Receptors as Drug Target for Treatment of Anxiety and Depression

### 4.1. Effect of Current Antidepressants on 5-HT3 Receptors

Some of the currently used antidepressants show affinity for 5-HT_3_ receptors. Thus, the tricyclic antidepressant (TCA) Imipramine, the SSRI fluoxetine, the non-selective α2-adrenoceptor antagonist mirtazapine and the MAOI phenelzine dose-dependently block the inward current mediated by 5-HT_3_ receptors expressed in cultured cells [152-156]. Moreover, several antidepressants, including fluoxetine, can inhibit binding of a 5-HT_3_ receptor antagonist [157]. Interestingly, fluoxetine induced an increase of Polysialic Acid Neural Cell Adhesion Molecule (PSA-NCAM), a molecule implicated in neuroplasticity, an effect that was blocked by administration of 5-HT_3_ receptor antagonist, thus suggesting a direct effect of fluoxetine on 5-HT_3_ receptors [158]. Finally, fluoxetine inhibits 5-HT release induced by 5-HT_3_ receptor agonists in the dorsal raphe nucleus [78]. Except for mirtazapine, the latter effect of antidepressants on 5-HT_3_ receptors seems non-competitive[155,156,159]. Similarly, typical and atypical antipsychotics antagonize 5-HT_3_ receptor in a non competitive manner [160]. This has led to the suggestion of an allosteric recognition site different from the 5-HT_3_ binding site [155]. Interestingly, the non-competitive 5-HT3 antagonism of antidepressants seem not associated with an increase of internalization [161]. It has also been shown that 5-HT_3_ receptors and antidepressants are colocalized in specific domain of membrane cells, the raft-like domains. Nevertheless antidepressants may exert effect on 5-HT_3_ receptor despite disruption of lipid rafts [161,162].

Electrophysiological studies exploring recovery of firing after chronic (from 2 days to 3 weeks) antidepressant treatments in anesthetized rats suggested that mirtazapine may be more rapid than SSRIs in reversing the decrease of 5-HT dorsal raphe nucleus firing rate. Also, co-administration of paroxetine and mirtazapine may accelerate this index of antidepressant response compared to either drug alone [163]. In preclinical studies, cyamemazine, an atypical antipsychotic with D2-like, 5-HT_2A_, 5-HT_2C_ and 5-HT_3_ receptor antagonism properties [164] presents anxiolytic-like activity. In clinical studies, cyamemazine improved the anxious syndrome [164]. Finally, it has been suggested that electroconvulsive therapy may potentiate 5-HT_3_ receptor function in hippocampal CA1 pyramidal cells [165].

### 4.2. Lu AA21004

Lu AA21004 is a 5-HT_3_ receptor antagonist, 5-HT_1A_ receptor agonist (h5-HT3 and h5-HT_1A_ receptors: Ki = 4.5 and 15 nM, respectively) and an inhibitor of the 5-HT transporter (5HTT) (h5HTT: IC_50_ = 5.4 nM) [166]. This *in vitro* profile translates into enhanced levels of 5-HT as well as other neurotransmitters (*i.e.*, noradrenaline, dopamine, acetylcholine) in hippocampus and prefrontal cortex after *in vivo* administration [91,166]. It is different from others antidepressants since it has an effect on 5-HT levels even at a low transporter occupancy [91,166]. Moreover, it displays anxiolytic-like and antidepressant-like activity in various validated rodents models with a better efficacy than current antidepressant drugs [91,166]. Interestingly, in electrophysiological studies, Lu AA21004 has a preclinical profile of a fast-acting antidepressant, since it induced early 5-HT_1A_ receptor desensitization compared to fluoxetine, an effect probably mediated by 5-HT_3_ receptors blockade [167]. Currently in phase III trials, LuAA21004 displays very good efficacy and well tolerance [168].

### 4.3. Preclinical Studies of 5-HT_3_ Receptor Ligands

Poncelet *et al.* [169] demonstrate that the selective 5-HT_3_ receptor agonist SR57227A produces antidepressant-like effect in different behavioral tests (forced swimming test, learned helplessness) in rodents. In contrast, other investigators report 5-HT_3_ receptor agonists alone or in combination with antidepressants to be ineffective in the forced swimming test [170] and others report that 5-HT_3_ receptor agonists attenuate the effects of antidepressants in this animal model [171]. These variable results may be explained by the different doses tested and may also ascribed to SR57227A being a partial 5HT_3_ agonist.

In the learned helplessness test, zacopride, ondansetron and tropisetron reverse the escape failure with a biphasic dose effect relationship [70]. In the forced swim test and tail suspension test, acute and chronic treatments with 5-HT_3_ receptor antagonists decrease the immobility time [71,172-175]. Interestingly, in the forced swim test, ondansetron increases the efficacy of fluoxetine, venlafaxine and citalopram [71,170]. In bulbectomized rats (another animal model of depression) 5-HT_3_ receptor antagonists reverse their depression-like phenotype [71,175]. The novel putative antidepressant (4-benzylpiperazin-1-yl) (quinoxalin-2-yl) methanone (QCF-3) which is a 5-HT_3_ receptor antagonist displayed also antidepressant properties both alone and in association with fluoxetine [176]. Finally, antidepressants induce a decrease in rapid eyes movements (REM) sleep. Microinjection of the 5-HT_3_ receptor agonist mCPBG in the dorsal raphe nucleus causes a reduction of rapid eye movement sleep (REMS) whereas ondansetron prevented this reduction. [150]. Thus, administration of a 5-HT_3_ receptor antagonist may have a beneficial effect on sleep disturbances induced by antidepressants.

Anxiolytic activity of 5-HT_3_ receptor antagonists have been extensively studied in animal models [177,178]. Griebel *et al.* [178] analyzed more than 75 5-HT_3_ receptor related experiments in which two-third of the studies demonstrated anxiolytic-like effect of 5-HT_3_ receptor antagonists and one-third of the studies failed to reveal an effect. For example, in some studies, 5-HT_3_ receptor antagonists disinhibited punished behavior in the Vogel test [145,179,180] and in other studies 5-HT_3_ receptor antagonists were inactive [181]. Interestingly, after chronic treatment with diazepam, 5-HT_3_ receptor agonists had no more anxiogenic effect, suggesting that diazepam may have induced a 5-HT_3_ receptor desensitization [182].

Another index used to evaluate anxiolytic effect of drugs is by examining its effect on cholecystokinin [183]. Interestingly, GABAergic interneurons expressing 5-HT_3_ receptors co-express cholecystokinin [88,104]. It has been shown that 5-HT or 5-HT_3_ receptor agonists induce a release of cholecystokinin in cortical or accumbal synaptosomes while 5-HT_3_ receptor antagonists decrease spontaneous- or induced-release of CCK [184,185]. However, in another study, 5-HT_3_ receptor antagonists fail to prevent the increase of CCK induced by stress in rat prefrontal cortex [186]. Finally, it is well known that amygdala is involved in physiology of anxiety [187]. As described previously, 5-HT_3_ receptors are present at significant levels in the amygdala. In the mouse amygdala, local administration of 5-HT_3_ receptor antagonists attenuates aversive response whereas 5-HT_3_ receptor agonists increase this [146]. Under the same conditions, in the social interaction test, 5-HT_3_ receptor antagonists have also an anxiolytic-like activity [146]. It has been suggested that the amygdala is involved in disinhibitory effects induced by various 5-HT_3_ receptor antagonists [188].

5-HT_3A_ receptor knockout male mice present anxiety-like behavior in elevated plus maze, novelty interaction animal models and light/dark box models of anxiety [189,190]. In the forced swim test, male 5-HT_3A_ knock-out (KO) did not differ from the wild type rats, whereas female 5-HT_3_ knockout mice showed increased immobility [191]. Mice overexpressing 5-HT_3_ receptors show decreased anxiety in the elevated plus-maze and in the exploration paradigm [192].

### 4.4. Clinical Studies with 5-HT_3_ Receptor Antagonists

5-HT_3_ receptor antagonists have been tested on anxiety and depressive syndromes associated to other diseases. For example, ondansetron reduced depressive symptoms in patients with chronic hepatitis C [193], with alcoholism [194] and in bulimic patients [195]. Similarly in fibromyalgic patients, tropisetron improved anxiety and depressive scores [196]. Moreover, in pathology with common symptoms of depression like chronic fatigue or fibromyalgia, 5-HT_3_ receptor antagonist have been reported efficacious [193,197-199], although negative results in chronic fatigue syndrome have been also reported [200]. Suspicion of 5-HT syndrome after use of setron alone or in combination with mirtazapine has reported in one study [201].

In healthy volunteers, ondansetron has been explored in emotional processing tasks. It abolishes the emotion potentiated startle effect, revealing an anxiolytic-like activity [202]. In anxious patients, zatosetron tends to reduce anxiety however the results from this study was not significant [203]. Similarly, tropisteron improves anxiety scores in patients suffering from generalized anxiety disorders [204]. In clinical studies, results concerning CCK release were contradictory as in preclinical studies. Indeed, in patients with panic disorder and social phobia, ondansetron fails to prevent the anxiety induced by pentagastrin, a CCK agonist [205]. Whereas, in healthy volunteers, ondansetron prevents panic symptoms induced by CCK tetrapeptide [206].

## 5. Conclusions

5-HT_3_ receptors are in numerous ways intimately involved in the regulation of neurotransmitter systems that are known to be of relevance for several psychiatric disorders including mood disorders, schizophrenia, eating disorders and addiction [21,207]. As becomes evident from the present review, the involvement of 5-HT_3_ receptors is complex and also context dependent. Their molecular structure, function and regulation are only partly elucidated. It will be important to understand why several responses associated with 5-HT_3_ receptor ligands present a bell-shaped dose-response curve. In conclusion, we feel that additional knowledge about 5-HT_3_ receptor function and their role in several diseases may offer new therapeutic opportunities in the future.

## Figures and Tables

**Table 1:** 5-HT_3_ receptor agonists and antagonists.

**5-HT3 receptor agonist**	**5-HT3 receptor antagonists**
1-(m-chlorophenyl)-biguanide (mCPBG)	MDL 73,147EF (dolasetron)
2-methyl-5-HT (2-me-5-HT) SR57227A	GR38032F (ondansetron)
	BRL 43694 (granisetron)
	ICS 205-939 (tropisetron)
	DAU 6215 (itasetron)
	BRL-46470A (ricasetron)
	LY277359 (zatosetron)
	MDL 72222
	Palanosetron

**Table 2:** Acute and chronic effects of 5-HT_3_ receptors antagonists on the number of DA cell/track. 5-HT3 receptor antagonists had either no effect (0) either an increase (+) or a decrease (−) on the number of cell/track.

**Cell per track**				
**5-HT_3_ receptor antagonist**	**Duration**	**Dose**	**A9 (SNc)**	**A10 (VTA)**
Dolasetron [117]	Acute treatment	500 µg/kg, i.v.	0	0
Zatosetron [116]		0.1 mg/kg, i.v.	0	+
		1 mg/kg, i.v.	0	+
Zatosetron [147]		10 mg/kg, i.v.	0	0
		0.01 mg/kg, i.p.	0	0
		0.1 mg/kg, i.p.	0	−
		1 mg/kg, i.p.	0	0
		10 mg/kg, i.p.	0	0
Itasetron [118]		15 µg/kg, s.c.	0	+
Dolasetron [117]	Chronic treatment (21 days)	5 mg/kg/day, i.p.	−	−
Granisetron [137]		5 mg/kg/day, i.p.	0	0
		10 mg/kg/day, i.p.	0	0
Itasetron [118]		30 µg/kg/day, s.c.	0	−
Zatosetron [116]		0.1 mg/kg/day, i.p.	−	−
		1 mg/kg/day, i.p.	0	+
		10 mg/kg/day, i.p.	0	−
Zatosetron [147]		0.01 mg/kg, i.p.	0	0
		0.1 mg/kg, i.p.	0	−
		1 mg/kg, i.p.	0	0
		10 mg/kg, i.p.	0	0
